# Alleviation effect of glycyrrhetinic acid on zearalenone-induced reproductive toxicity in replacement gilts

**DOI:** 10.3389/fvets.2025.1636738

**Published:** 2025-10-13

**Authors:** Li-Tao Che, Ahmed H. El-Sappah, Heba Allah M. Elbaghdady, Yong-Jun Ren, Qi-Fan Wu, Wan-Hai Zhou, Ya-ru Yang, Rong-Fu Guo

**Affiliations:** ^1^Faculty of Agriculture, Forestry and Food Engineering, Yibin University, Yibin, China; ^2^Yibin Inspection and Quarantine Engineering Technology Research Center of Animal and Plant, Yibin University, Yibin, China; ^3^Geneics Department, Faculty of Agriculture, Zagazig University, Zagazig, Egypt; ^4^Department of Zoology, Faculty of Science, Mansoura University, Mansoura, Egypt; ^5^Sichuan Animal Science Academy, Chengdu, China; ^6^Animal Breeding and Genetics Key Laboratory of Sichuan Province, Chengdu, China; ^7^Yunnan Provincial Key Laboratory of Animal Nutrition and Feed Science, Yunnan Agricultural University, Kunming, China; ^8^Faculty of Animal Science and Technology, Yunnan Agricultural University, Kunming, China

**Keywords:** glycyrrhetinic acid (GA), zearalenone (ZEN), reproductive hormones, reproductive organs, Estrus onset, hydroxysteroid dehydrogenase

## Abstract

**Introduction:**

This study investigated whether glycyrrhetinic acid (GA) can alleviate the reproductive toxicity of Zearalenone (ZEN) in replacement gilts.

**Methods:**

Eighty Landrace × Yorkshire gilts were randomly assigned to four dietary groups: control (basal diet), ZEN (1 mg/kg), GA (400 mg/kg), and ZEN + GA (1 mg/kg ZEN + 400 mg/kg GA).

**Results:**

The onset of estrus advanced significantly in all treatment groups, with the GA and ZEN + GA groups showing the most pronounced changes. Puberty onset occurred earlier in the ZEN group and was further advanced by GA supplementation. ZEN exposure impaired uterine and ovarian development, while GA improved organ development and mitigated the abnormalities in the ZEN + GA group. Hormonal analysis revealed that ZEN reduced estradiol (E2) and luteinizing hormone (LH), whereas GA elevated all measured hormones. The ZEN + GA group showed a partial recovery in hormone levels, excluding E2. Histological examination of liver tissue in the ZEN group revealed focal hepatocellular necrosis and lymphocyte infiltration, which GA notably attenuated. ZEN upregulated 3α/3β/17β-hydroxysteroid dehydrogenase (*HSD*) gene expression in the liver and duodenum, while GA co-administration downregulated most *HSD* genes except hepatic *3α-HSD*.

**Discussion and conclusion:**

These findings suggest that GA can alleviate ZEN-induced reproductive toxicity via modulation of endocrine and hepatic metabolic pathways.

## 1 Introduction

The reproductive performance of sows is a key factor determining the level of pork production and its economic benefits globally. To improve sow performance, interventions should also target the health status of replacement gilts. For instance, poor nutrition or management can cause anestrus. In intensive production systems, this can affect up to 30% of replacement gilts, and the proportion of multiparous sows culled due to failed estrus initiation has been reported to be as high as 40% (1). Zearalenone (ZEN) is a non-steroidal fungal toxin with estrogenic activity produced by *Fusarium* species that has been shown to cause serious reproductive harm to animals, especially replacement gilts (2). Although preventing the production of mycotoxins is the best way to eliminate contamination, most molds are environmentally persistent, and contamination of feed is challenging to avoid throughout the entire process from field to storage to processing (3). Currently, the methods for detoxifying mycotoxins mainly include a combination of physical, chemical, and biological processes, but there are concerns regarding their safety and effectiveness (4–7).

ZEN enters the body through ingestion of contaminated feed or milk and undergoes hydroxylation biotransformation under the action of 3*α*-steroid dehydrogenase (3α-HSD) and 3*β*-steroid dehydrogenase (3β-HSD), primarily in the intestine and liver, into ɑ- and β-zearalenol (ɑ/β-ZEL), which are metabolites of ZEN in maize. In pigs and ruminants, small amounts of ZEN and some *α*/*β*-ZEL can be further reduced to ɑ- and *β*-zearalanol (ɑ/β-ZAL) (7, 8). The affinity of α-ZEL for the estrogen receptor (ER) is 500 times that of ZEN. The relative binding affinity of ZEN and its metabolites to ER is as follows: α-ZEL > α-ZAL > β-ZEL > ZEN > β-ZAL (4). The toxicity of ZEN and its metabolites may be especially increased in sensitive animals, such as pigs.

Glycyrrhetinic acid (GA), an extract from herbs, acts both as a plant estrogen that regulates the reproductive functions of animals in a bidirectional manner (9) and also serves as an inhibitor of key steroid dehydrogenases involved in the generation and metabolism of steroid hormones, including 3α-HSD, 3β-HSD, and 17β-steroid dehydrogenase (17-HSD) (10, 11). It was known that GA can affect the reproductive toxicity of ZEN by regulating its metabolism in animals and the circulating levels of endocrine hormones (12). A previous study demonstrated that adding 400 mg/kg GA to the feed of replacement gilts affected their serum levels of reproductive hormones and alleviated the effects of ZEN on vaginal development (12). So far, there have been few reports on the effects of GA on estrus and reproductive organ development in gilts. We aimed to investigate the detrimental effects of adding ZEN to feed on replacement gilts, and to study the effects of adding GA to feed on estrus, uterine, and ovarian follicle development. This study explored the effects of GA on mitigating the harm caused by ZEN to the reproductive performance of replacement gilts. It investigated its underlying mechanism, providing new ideas for improving the reproductive potential and productivity of replacement gilts.

## 2 Materials and methods

### 2.1 Ethics statement

All experimental procedures used in this study were approved by the Ethics Committee of Life Sciences of Yibin University (Approval Number: YBECS-2022-0015, Date of approval: March 9, 2022).

### 2.2 Materials

Glycyrrhetinic acid (GA, purity ≥ 98%) was purchased from Nanjing Zelang Pharmaceutical Technology Co., Ltd. (Nanjing, China), and Zearalenone (ZEN, purity ≥ 98%) was purchased from Sigma Company (Germany).

### 2.3 Experimental design

Eighty healthy and well-conditioned Landrace × Yorkshire (LY) replacement gilts (average age 165 ± 7 days, average body weight (BW) 94.05 ± 3.82 kg) were randomly divided into four groups (*n* = 20/group) with five replicates per group and four gilts per replicate. The gilts were fed either a basal diet (control) or a basal diet supplemented with ZEN or GA. Replacement gilts in the control group were fed basal diets, ZEN, GA, and ZEA + GA groups were fed basal diets supplemented with ZEN (1 mg/kg), GA (400 mg/kg), and compound additives (1 mg/kg ZEN + 400 mg/kg GA). After a 5-day acclimation period, all gilts were fed the prescribed diet until the 19th day of their second estrus period. The experimental gilts were housed in one unit per replicate. The gilts were fed a restricted diet (calculated as 2.5 kg/day per head), and all gilts had free access to water throughout the entire period. Other breeding management and immunization procedures were uniformly implemented according to the regulations of the pig farm.

### 2.4 Experimental diet

The basal diet (Table 1) was formulated according to the National Research Council (40). The feed was prepared once before the experiment, and an antiseptic was added to the premix. The experimental feed was packed and stacked on wooden pallets and stored in a clean, cool, dry, and well-ventilated place. Enzyme-linked immunosorbent assay (ELISA) was used to detect the contents of ZEN, Vomitoxin (DON), and Aflatoxin B1 (AFB1) in the feed of each group. The contents of ZEN in each diet were 38.30, 1032.764, 31.41, and 1028.92 μg/kg, and the contents of DON and AFB1 in each diet all met the limit requirements of China’s National Feed Sanitation Standard (GB 13078–2017).

**Table 1:** Composition and nutrient levels of basal diet (%, air-dry basis).

Component	Content, %	Nutrients	Value, %
Corn	68.85	Dry matter %	85.82
Soybean meal	17.00	Digestible energy, MJ/kg	13.06
Wheat bran	10.00	Crude protein	14.88
Limestone石粉	1.02	Ash	5.23
CaHPO_4_	1.58	Ether extract	3.18
NaCl	0.30	Crude Fiber	2.79
L-Lysine HCl	0.17	Nitrogen free extract	59.74
Choline	0.08	Calcium	0.83
PremIX^1^	1.00	STTD phosphorus	0.65
Total	100.00	ATTD phosphorus	0.41
		Lysine	0.78
		Methionine	0.23
		Methionine+Cysteine	0.50
		Tryptophane	0.16
		Threonine	0.52

^1^Supplied per kilogram of diet: vitamin A, 12000 IU; vitamin D_3_, 2,700 IU; vitamin E, 48 mg; vitamin K_3_, 2 mg; vitamin B_1_, 2 mg; vitamin B_2_, 5.4 mg; vitamin B_6_, 2 mg; vitamin B_12_, 0.02 mg; folic acid, 1.5 mg; D-calcium pantothenate, 13.5 mg; niacin, 20 mg; biotin, 0.3 mg; Fe (FeSO_4_. H_2_O), 150 mg; Cu (CuSO_4_.5H_2_O), 35 mg; Zn (ZnSO_4_. H_2_O), 120 mg; Mn (MnSO_4_ H_2_O), 60 mg; I (KIO_3_), 0.6 mg, Se(NaSeO_3_), 0.3 mg; STTD, standardized total tract digestible; ATTD, apparent total tract digestible. ^2^DE was a calculated value, while the others were measured values.

### 2.5 Sample collection

At the end of the experiment, at 8:00 a.m. on the 19th day of the second estrus period, 10 mL of blood was drawn from the anterior vena cava of one randomly selected gilt from each replicate and placed in a sterilized centrifuge tube. The tube was left at room temperature for 30 min and centrifuged at 1500 × g for 15 min. The supernatant was divided into Eppendorf tubes and stored at −20 °C for the detection of serum hormone indicators.

Subsequently, the gilts from each group post-blood collection (*n* = 20/group) were euthanized and slaughtered using standard humane procedures. The reproductive organs (including ovaries, uterus, uterine horns, cervix, and vagina) were dissected and weighed, followed by dimensional measurements. For liver sampling, the largest lobe was selected; a portion of the tissue was snap-frozen in liquid nitrogen for subsequent genetic analysis, while another segment was rinsed with physiological saline and fixed in 10% neutral buffered formalin for histopathological examination. The duodenum was flushed with physiological saline to remove luminal contents, then rapidly frozen in liquid nitrogen for genetic assays. All samples designated for molecular analysis were transferred to a − 70 °C ultra-low temperature freezer on the same day of collection for long-term storage until further processing.

### 2.6 Determination indicators and methods

#### 2.6.1 Determination of reproductive performance

At 9:30 a.m. and 4:00 p.m. every day, gilts were teased twice with boars for no less than 15 min each time. After the first estrus (based on the first standing reflex), the age, weight, and backfat thickness were recorded. The time of initial estrus was documented, and then gilts were continuously fed until the 19th day of the second estrus period, whereupon weight and backfat thickness measurements were taken before slaughter. Total feed consumption was documented throughout the entire process, and the average daily weight gain (ADG), average daily feed intake (ADFI), and feed conversion ratio (F/G) were calculated.

#### 2.6.2 Measurement of uterine and ovarian parameters

Afterward, the reproductive organs (including ovaries, uterus, uterine horn, cervix, and vagina) were quickly removed, weighed, and measured for size. The uterine tissues were dissected from the intestinal tract and weighed. The lengths of the left uterine horn, right uterine horn, left fallopian tube, and right fallopian tube were measured. The ovaries were dissected from the termini of the fallopian tubes and weighed, and the number of large follicles (diameter > 5 mm) and medium-sized follicles (diameter ranging from 1 to 5 mm) was counted. Follicles were further evaluated and sorted by quality according to the following parameters:

(1) Healthy follicles: The follicular wall structure was complete, uniform, and tight, slightly pink or yellow, with evenly distributed capillaries and a bright red color. The follicular fluid was clear, and the cumulus was generally visible. (2) Early follicular atresia: The structure of the follicular wall was relatively uniform, but the inner layer was slightly flocculent, or there were a few dark patches on the wall. The follicle was slightly gray, white, with fewer or larger blood vessels, and a lighter color. The follicular fluid was slightly mixed, and a few cumulus cells were seen. (3) Late-stage follicular atresia: The structure of the follicular wall was uneven, with a dark color and few capillaries. The internal floc was severe, or dark lumps were seen inside the follicle. The cumulus was generally not visible (13, 14).

#### 2.6.3 Reproductive hormones measurements

An ELISA kit was used to measure serum estradiol (E_2_), insulin-like growth factor (IGF-1), luteinizing hormone (LH), and Kisspeptin (Kp). The kit was purchased from Shanghai Enzyme Linked Biotechnology Co., Ltd., and the manufacturer’s instructions were followed.

#### 2.6.4 Pathological analysis of liver sections

The largest hepatic lobe was chosen for sampling. The liver tissue was washed with saline solution and fixed in 10% formalin for subsequent tissue section analysis. The Liver samples were dehydrated in an ethanol series, embedded in paraffin, sectioned, and stained for microscopic examination of porcine hepatic tissue morphology.

### 2.7 HSD gene expression detection

After the gilts were slaughtered, a total of 40 samples of liver and duodenum were collected in sterile containers, snap-frozen in liquid nitrogen, and stored at −80 °C.

#### 2.7.1 Primer design

*β*-actin was selected as the internal reference gene, and primers for *β*-actin and target genes *3α-HSD*、3β-HSD, and 17β-HSD were synthesized by Shanghai Generay Biotech Co., Ltd. The RT-PCR primers were designed primarily based on the reference (15, 16), and the RT-PCR primer information is shown in Table 2.

**Table 2:** Information on primers used for RT-PCR.

Genes	Accession no.	Forward and reverse primers	Product length (bp)	Annealing temperature (°C)
*3α-HSD*	NM_001038626	5-GGATGCCAGTGGGAAAGTTAT	215	58
		5,-GAAGTTTGCTCTGATTGAGGTAAG-3		
*3β-HSD*	AF232699.2	5-TCCTGGCAAGTATTTCTCGG-3	108	57
		5-CCAGCAACAAGTGGACGAT-3		
*17β-HSD*	NM-001167649.1	5-ACAAACATCGCAGGCACG-3	280	60
		5-CAAATAGGACAGTAGGGCTAAATG-3		
*β-actin*	DQ845171.1	5-GGCCGCACCACTGGCATTGTCAT-3	104	60
		5-AGGTCCAGACGCAGGATGGCG-3		

#### 2.7.2 RNA extraction and gene expression detection

Total RNA was extracted from tissues using TRIzol Reagent according to the manufacturer^’s^ instructions (Invitrogen, Carlsbad, CA, United States), and genomic DNA was removed using DNase I (TaKara, Japan). RNA quality was determined using a 2,100 Bioanalyser (Agilent Technologies, Palo Alto, CA, United States) and quantified using an ND-2000 (Nano Drop Technologies, Wilmington, DE, United States). A high-quality RNA sample (OD_260/280_ = 1.8–2.2, OD_260/230_ ≥ 2.0, RIN ≥ 28.0, 28 S:18 S ≥ 1.0,>10 μg)was used to synthesize cDNA. Next, cDNA was synthesized from total RNA by the Reverse TIANScript II cDNA kit (Tiangen Biotech Co. Ltd., Beijing, China) and stored at- 20 °C. RT-PCR was conducted on Bio-Rad CFX96™ Real-Time PCR Systems (United States). The PCR conditions comprised initial denaturation at 95 °Cfor 10 min and 40 cycles of 95 °C for 5 s, 60 °Cfor 20s, and 72 °C for 15 s. The calculation method for the relative expression level of the target gene mRNA was based on the = 2^-△△Ct^ Ct method (17).

### 2.8 Statistical analysis

Data were analyzed by one-way ANOVA (SPSS22.0, IBM SPSS, Chicago), Duncan’s method was used for multiple comparisons between each group; Data were analyzed by 2 × 2 factorial analysis using SPSS 22.0 (IBM SPSS, Chicago) software. The results were expressed as the mean ± standard deviation. The *p* < 0.05 was considered statistically significant, and the *p* < 0.01 was considered highly significant.

## 3 Results

### 3.1 The effect of ZEN and GA on estrus onset in replacement gilts

The effect of ZEN and GA on estrus onset in replacement gilts was presented in Table 3. Analysis of the main effects showed that ZEN did not affect estrus onset indicators in replacement gilts, whereas GA had a highly significant effect on puberty weight in sows (*p* < 0.01) and significantly affected body weight on the 19th day of the second estrus period(Final BW), puberty age, and estrus interval in replacement gilts (*p* < 0.05). The interaction effects of ZEA and GA significantly affected the estrus interval in replacement gilts (*p* < 0.05).

**Table 3:** Effect of ZEN and GA on estrus onset in replacement gilts (*n* = 20/group).

Parameter		Initial BW, kg	Final BW, kg^1^	Puberty weight, kg	Puberty age, d	Puberty backfat, mm	Estrus interval, d
Groups	Control	94.17 ± 4.86	147.72 ± 9.10^a^	137.54 ± 5.27^Aa^	230.00 ± 10.55^Aa^	13.80 ± 1.17^a^	21.00 ± 1.26^b^
	GA	94.13 ± 3.5	135.06 ± 8.23^c^	123.36 ± 6.30^Bb^	195.00 ± 20.61^Bc^	12.17 ± 1.17^b^	21.00 ± 0.89^b^
	ZEN	93.97 ± 4.51	141.68 ± 9.69^b^	128.83 ± 7.10^Bb^	212.00 ± 16.98^ABb^	12.83 ± 0.75^b^	24.83 ± 3.75^a^
	ZEN + GA	93.93 ± 3.93	133.73 ± 9.26^c^	122.91 ± 7.69^Bb^	199.00 ± 28.25^Bc^	12.67 ± 0.82^b^	20.33 ± 0.82^b^
ZEA Adding Levels, mg/kg	0	94.15 ± 3.99	141.39 ± 10.56	130.45 ± 9.26	212.5 ± 24.05	12.99 ± 1.4	21.00 ± 1.03
	1	93.95 ± 3.99	137.71 ± 9.87	125.87 ± 7.64	205.50 ± 23.02	12.75 ± 0.96	22.58 ± 3.49
GA, Adding Levels, mg/kg	0	94.07 ± 4.42	144.7 ± 9.42^a^	133.19 ± 7.47^Aa^	221.00 ± 16.36^a^	13.32 ± 1.22	22.92 ± 3.32^a^
	400	94.03 ± 3.51	134.40 ± 8.29^b^	123.14 ± 6.63^Bb^	197.00 ± 23.41^b^	12.42 ± 0.99	20.67 ± 0.88^b^
*p* value	ZEA	0.917	0.378	0.143	0.448	0.637	0.107
	GA	0.983	0.022	0.004	0.017	0.086	0.027
	ZEA + GA	1.000	0.57	0.184	0.240	0.152	0.027

(1) Weight on the 19th day of the second estrus period. (2) In the same column, values with different small letter superscripts mean significant difference (*p* < 0.05), while values with different capital letter superscripts mean highly significant difference (*p* < 0.01). The same or no letter superscripts mean no significant difference (*p* > 0.05).

The results of one-way ANOVA showed that, compared with the control group, the Final BW of the ZEN group significantly advanced (*p* < 0.05), and the weight advancement was even more pronounced in the GA group and ZEN + GA group (*p* < 0.01). Compared with the control group, the puberty weight of the ZEN group, GA group, and ZEN + GA group replacement gilts was significantly advanced (*p* < 0.01). Compared with control gilts, all other groups had a significantly younger puberty age, and the puberty age of the GA group and ZEN + GA group was especially premature (*p* < 0.01). The pubertal backfat thickness of the replacement gilts was significantly reduced in all treatment groups compared with the control (*p* < 0.05), but there was no significant difference in backfat thickness among the ZEN, GA, and ZEN + GA groups (*p* > 0.05). The estrus interval of the ZEN group alone was significantly prolonged compared to that of the control, GA, and ZEN + GA groups (*p* < 0.05), and there was no significant difference in estrus interval among the control, GA, and ZEN + GA groups (*p* > 0.05). During the experiment, it was observed that some gilts in the ZEN group ended their estrus prematurely and experienced a relapse, resulting in disrupted estrus cycles.

### 3.2 The effect of ZEN and GA on serum reproductive hormones in replacement gilts

The effect of ZEN and GA on serum reproductive hormones in replacement gilts was presented in Table 4. Analysis of the main effects revealed that dietary supplementation with ZEN exerted highly significant effects on serum IGF-1 and E₂ concentrations in replacement gilts (*p* < 0.01), while GA supplementation significantly affected serum concentrations of IGF-1, kisspeptin (Kp), luteinizing hormone (LH), and estradiol (E₂; *p* < 0.01). The interaction between ZEN and GA showed highly significant effects (*p* < 0.01) on serum IGF-1, Kp, and E₂ concentrations, and significant effects (*p* < 0.05) on LH concentrations in replacement gilts.

**Table 4:** Effect of ZEN and GA on serum reproductive hormones in replacement gilts (*n* = 5/group).

Parameter		IGF-1 (ng/ml)	Kp (pg/ml)	LH (mIU/ml)	E_2_ (pg/ml)
Groups	Control	78.89 ± 3.70^Cd^	1233.83 ± 182.38^Bc^	9.15 ± 1.24^Bb^	41.37 ± 7.55^Bb^
	GA	211.93 ± 9.35^Aa^	1707.15 ± 166.51^Aa^	12.12 ± 1.62^Aa^	67.33 ± 4.58^Aa^
	ZEN	160.61 ± 5.865^B^	1378.88 ± 114.0^Bb^	5.66 ± 0.44^Cc^	27.35 ± 3.40^Cc^
	ZEN + GA	181.02 ± 3.57^Bb^	1384.90 ± 161.29^Bb^	12.46 ± 2.68Aa	37.44 ± 2.55^Bb^
ZEA Adding Levels, mg/kg	0	145.41 ± 70.44^Bb^	1470.49 ± 298.89	10.64 ± 2.07	54.35 ± 14.89^Aa^
	1	170.82 ± 11.29^Aa^	1381.89 ± 131.71	9.06 ± 4.02	32.4 ± 6.03^Bb^
GA, Adding Levels, mg/kg	0	119.75 ± 43.21^Bb^	1306.36 ± 162.491^Bb^	7.41 ± 2.04^Bb^	34.36 ± 9.22^Bb^
	400	196.48 ± 17.60^Aa^	1546.03 ± 229.63^Aa^	12.29 ± 2.101^Aa^	52.39 ± 16.14^Aa^
*p* value	ZEA	<0.001	0.228	0.055	<0.001
	GA	<0.001	0.004	<0.001	<0.001
	ZEA + GA	<0.001	0.004	0.023	0.002

(1) Weight on the 19th day of the second estrus period. (2) In the same column, values with different small letter superscripts mean significant difference (*p* < 0.05), while values with different capital letter superscripts mean highly significant difference (*p* < 0.01). The same or no letter superscripts mean no significant difference (*p* > 0.05).

The results of one-way ANOVA showed that, compared with the control group, the levels of IGF-1 in the ZEN group, GA group, and ZEN + GA group were significantly increased (*p* < 0.01), and this effect was most pronounced in the GA and ZEN + GA groups (*p* < 0.01). Likewise, compared with control, the levels of kisspeptin (Kp) in all treatment groups were significantly increased (*p* < 0.05), and this was most pronounced in the GA group (*p* < 0.01). Compared with the control group, the LH serum concentration in the ZEN group was significantly reduced (*p* < 0.01), while in the GA group and ZEN + GA group, it was significantly increased (*p* < 0.01). Finally, E_2_ serum concentration was significantly reduced in the ZEN group compared with the control (*p* < 0.01) and significantly increased in the GA group (*p* < 0.01). There was no significant difference in E_2_ between the ZEN + GA group and the control group (*p* > 0.05), but it was significantly higher than that in the ZEN group.

### 3.3 The effect of ZEN and on the development of the uterus and ovarian follicles in replacement gilts

The effect of ZEN on the development of the uterus and ovarian follicles in replacement gilts was presented in Table 5. Analysis of the main effects revealed that dietary ZEA supplementation exerted highly significant effects (*p* < 0.01) on uterine weight, ovarian weight, left fallopian tube length, number of large follicles, number of medium follicles, and number of healthy mature follicles in replacement gilts. Dietary GA supplementation significantly affected ovarian weight, left and right fallopian tube lengths, number of medium follicles, and number of healthy mature follicles (*p* < 0.01), whereas it exerted a significant effect (*p* < 0.05) on the number of large follicles. The interaction between ZEA and GA demonstrated highly significant effects (*p* < 0.01) on uterine weight, number of large follicles, number of medium follicles, and number of healthy mature follicles.

**Table 5:** Effects of ZEN and GA on the development of the uterus and ovarian follicles in replacement gilts (*n* = 5/group).

Parameter		Uterine weight, g	Ovarian weight, g	Left uterine horn length, cm	Right uterine horn length, cm	Left fallopian tube length, cm	Right fallopian tube length, cm	Number of large follicles (diameter> 5 mm)	Number of medium follicles (diameter 1–5 mm)	Number of healthy mature follicles
Groups	Control	666.67 ± 42.19^C^	16.33 ± 2.0^ABb^	127.00 ± 15.21^Bc^	130.00 ± 13.42^Bc^	26.50 ± 4.02	30.35 ± 2.37	18.00 ± 3.00^Aa^	43.67 ± 3.21^Aa^	18.00 ± 3.00^Aa^
	GA	763.17 ± 45.81^Bb^	19.67 ± 7.28^Aa^	161.67 ± 32.52^Aa^	157.00 ± 21.48^Aa^	25.83 ± 4.06	27.00 ± 0.89	17.33 ± 3.53^Aa^	43.00 ± 2.00^Aa^	17.33 ± 3.53^Aa^
	ZEN	889.00 ± 63.90^Aa^	11.00 ± 2.68^Cc^	99.67 ± 9.40^Cd^	113.00 ± 18.26^Bc^	30.00 ± 3.49	29.47 ± 3.42	8.00 ± 3.00^Bb^	24.67 ± 2.53^Bb^	2.00 ± 1.26^Bb^
	ZEN + GA	724.00 ± 60.40^Bb^	15.67 ± 4.52^Bb^	148.67 ± 21.47^ABb^	148.00 ± 14.20^ABb^	25.70 ± 2.06	30.10 ± 6.17	16.33 ± 3.57^Aa^	41.00 ± 2.00^Aa^	16.33 ± 3.57^Aa^
ZEA Adding Levels, mg/kg	0	714.92 ± 65.65^Bb^	18 ± 2.84^Aa^	144.34 ± 21.82^Aa^	143.5 ± 22.08	26.17 ± 3.83	28.68 ± 2.44	17.67 ± 3.11^Aa^	43.34 ± 2.55^Aa^	17.67 ± 3.11^Aa^
	1	806.5 ± 104.87^Aa^	13.34 ± 3.53^Bb^	124.17 ± 27.3^Bb^	130.5 ± 24.04	27.85 ± 3.53	29.79 ± 4.71	12.17 ± 5.38^Bb^	32.84 ± 8.87^Bb^	9.17 ± 7.967^Bb^
GA, Adding Levels, mg/kg	0	777.84 ± 127.81	13.67 ± 3.59^Bb^	113.34 ± 18.7^Bb^	121.5 ± 17.56^Aa^	28.25 ± 4.00	29.91 ± 2.81	13.00 ± 5.98^b^	34.17 ± 10.38^Bb^	10.00 ± 8.717^Bb^
	400	743.59 ± 54.59	17.67 ± 3.29^Aa^	155.17 ± 11.2^Aa^	152.5 ± 17.81^Bb^	25.77 ± 3.04	28.55 ± 4.47	16.83 ± 3.39^a^	42.00 ± 2.16^Aa^	16.83 ± 3.39^Aa^
*p* value	ZEA	0.002	<0.001	<0.001	0.109	0.298	0.517	0.002	<0.001	<0.001
	GA	0.174	0.003	<0.001	<0.001	0.132	0.429	0.019	<0.001	<0.001
	ZEA + GA	<0.001	0.564	0.170	0.609	0.264	0.252	0.007	<0.001	<0.001

(1) Weight on the 19th day of the second estrus period. (2) In the same column, values with different small letter superscripts mean significant difference (*p* < 0.05), while values with different capital letter superscripts mean highly significant difference (*p* < 0.01). The same or no letter superscripts mean no significant difference (*p* > 0.05).

The results of one-way ANOVA showed that, compared with the control group, the weight of the uterine body of the ZEN, GA, and ZEN + GA groups increased significantly (*p* < 0.01). Still, the most significant mean uterine body weight was from the ZEN group (*p* < 0.01). By contrast, compared with the control, the ovarian weight of the ZEN group decreased significantly (*p* < 0.01), while the ovarian weight of the GA group increased significantly (*p* < 0.05). There was no significant difference in ovarian weight between the ZEN + GA group and the control group (*p* > 0.05), but it was significantly higher than that of the ZEN group (*p* < 0.01). Consistently, compared with the control, the weight of the left uterine horn was significantly reduced in the ZEN group (*p* < 0.05). In contrast, the weights of the left and right uterine horns in the GA and ZEN + GA groups were significantly increased (*p* < 0.01). The left uterine horn in the GA and ZEN + GA groups was significantly heavier than that in the ZEN group (*p* < 0.01). There was no significant difference in the length of the left and right fallopian tubes among the groups (*p* > 0.05). Compared with the control group, the number of large follicles, medium follicles, and healthy mature follicles in the ZEN group was significantly reduced (*p* < 0.01), while there was no significant difference between the GA group, ZEN + GA group, and the control group (*p* > 0.05). The number of large follicles, medium follicles, and healthy mature follicles in the GA and ZEN + GA groups was significantly higher than that in the ZEN group (*p* < 0.01).

### 3.4 The effect of ZEN and GA on the external structures of the uterus in replacement gilts

**Figure 1:**
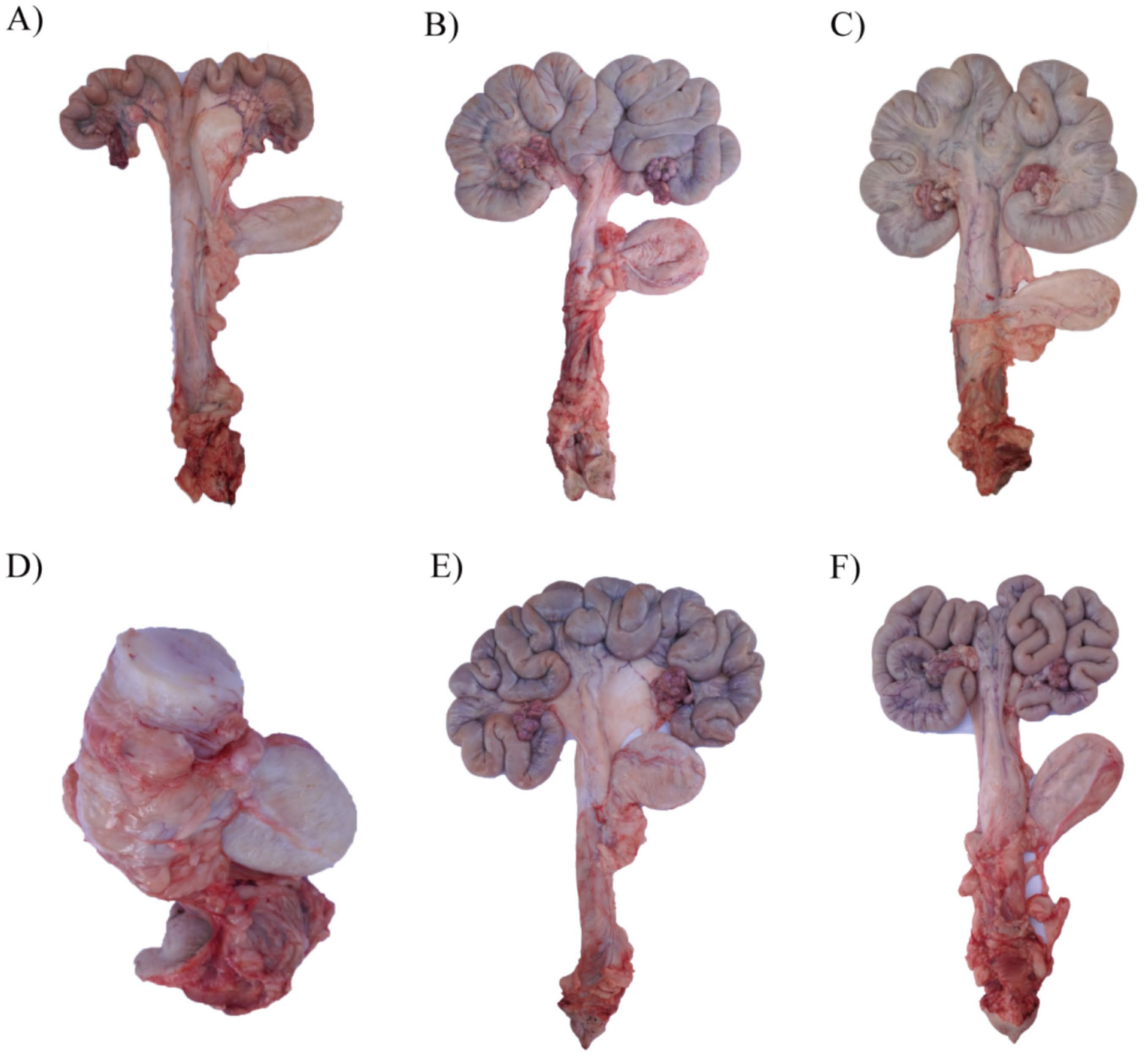
Effect of ZEN and GA on uterine development in replacement gilts: **(A)** A uterus from a gilt in the control group pre-estrus. **(B)** A uterus from a gilt in the control group on the 19th day of the second estrus. **(C,D)** Two representative uteri from gilts in the ZEN group on the 19th day of the second estrus. **(E)** A uterus from a gilt in the GA group on the 19th day of the second estrus. **(F)** A uterus from a gilt in the ZEA + GA group on the 19th day of the second estrus.

As shown in Figure 1, compared with the control, gilts in the ZEN group had enlarged and shortened uterine horns and a thickened and enlarged cervix (Figures 1A–C). During the experiment, it was found that some gilts in the ZEN group developed significant hardening of the cervix and cartilage formation (Figure 1D). Compared with other groups, the uterine horns in the GA group were significantly longer, and the number of loops increased (Figure 1E). In the ZEN + GA group, the uterine horns were also significantly longer with increased loops, but the uterine horns were much thinner (Figure 1F).

### 3.5 The effect of ZEN and GA on ovarian follicles in replacement gilts

**Figure 2:**
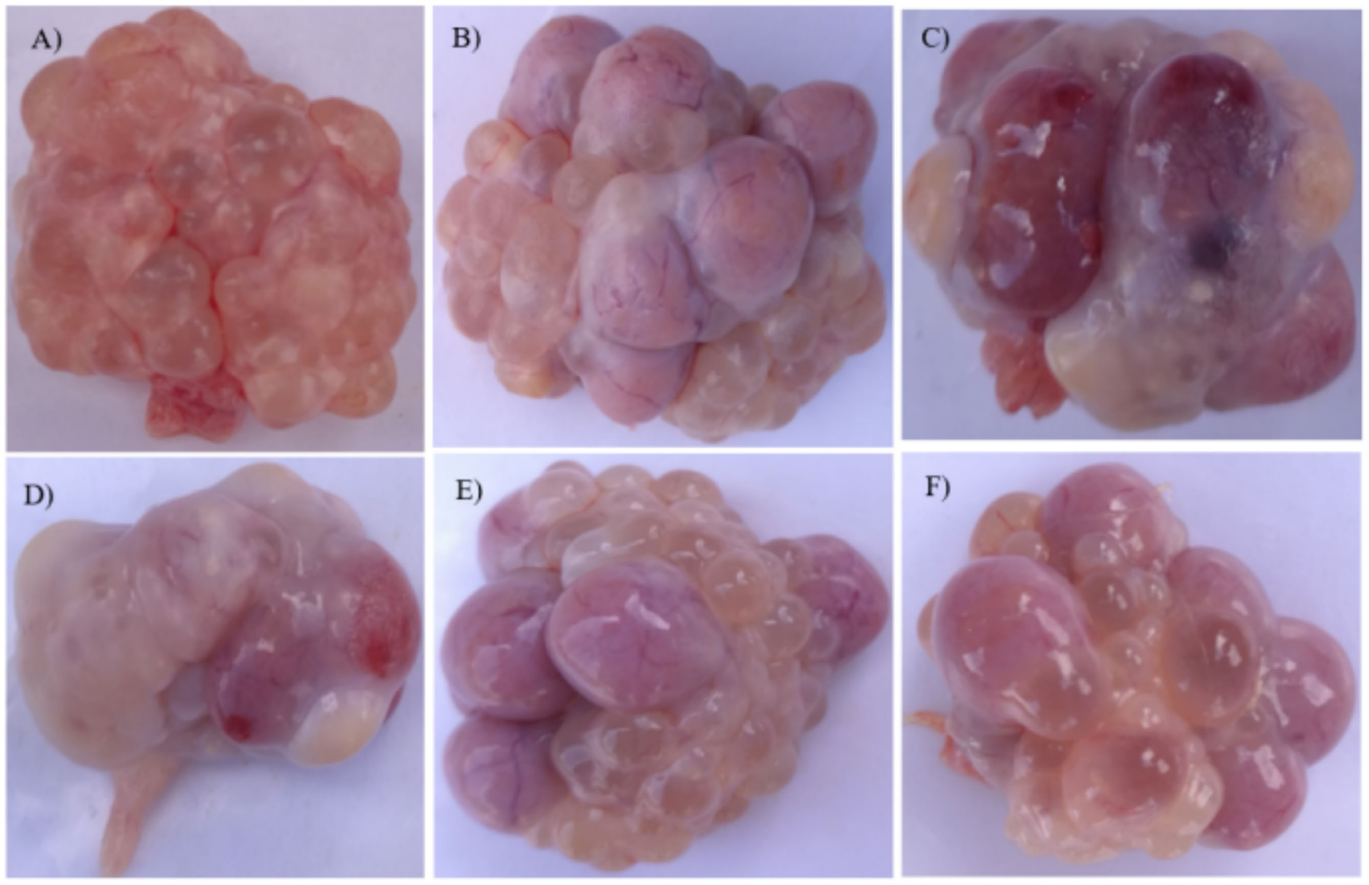
Effect of ZEN and GA on ovarian follicles t in replacement gilts: **(A)** Ovaries from replacement gilts pre-estrus in the control group. **(B)** Ovaries from the control group were taken on the 19th day of the second estrus. **(C,D)** Ovaries from the ZEN group taken on the 19^th^ day of the second estrus. **(E)** Ovaries from the GA group were taken on the 19th day of the second estrus. **(F)** Ovaries from the ZEN + GA group were taken on the 19th day of the second estrus.

The ovarian and follicular of replacement gilts in the control, GA, and ZEN + GA groups was normal (Figures 2A,B,E,F). However, gilts in the ZEN group had abnormal ovarian development, with atrophy of the ovaries, the appearance of blackening, whitening, and yellowing of follicles, and indistinct borders between follicles (Figures 2C,D).

### 3.6 Effect of ZEN and GA on the liver of replacement gilts

**Figure 3:**
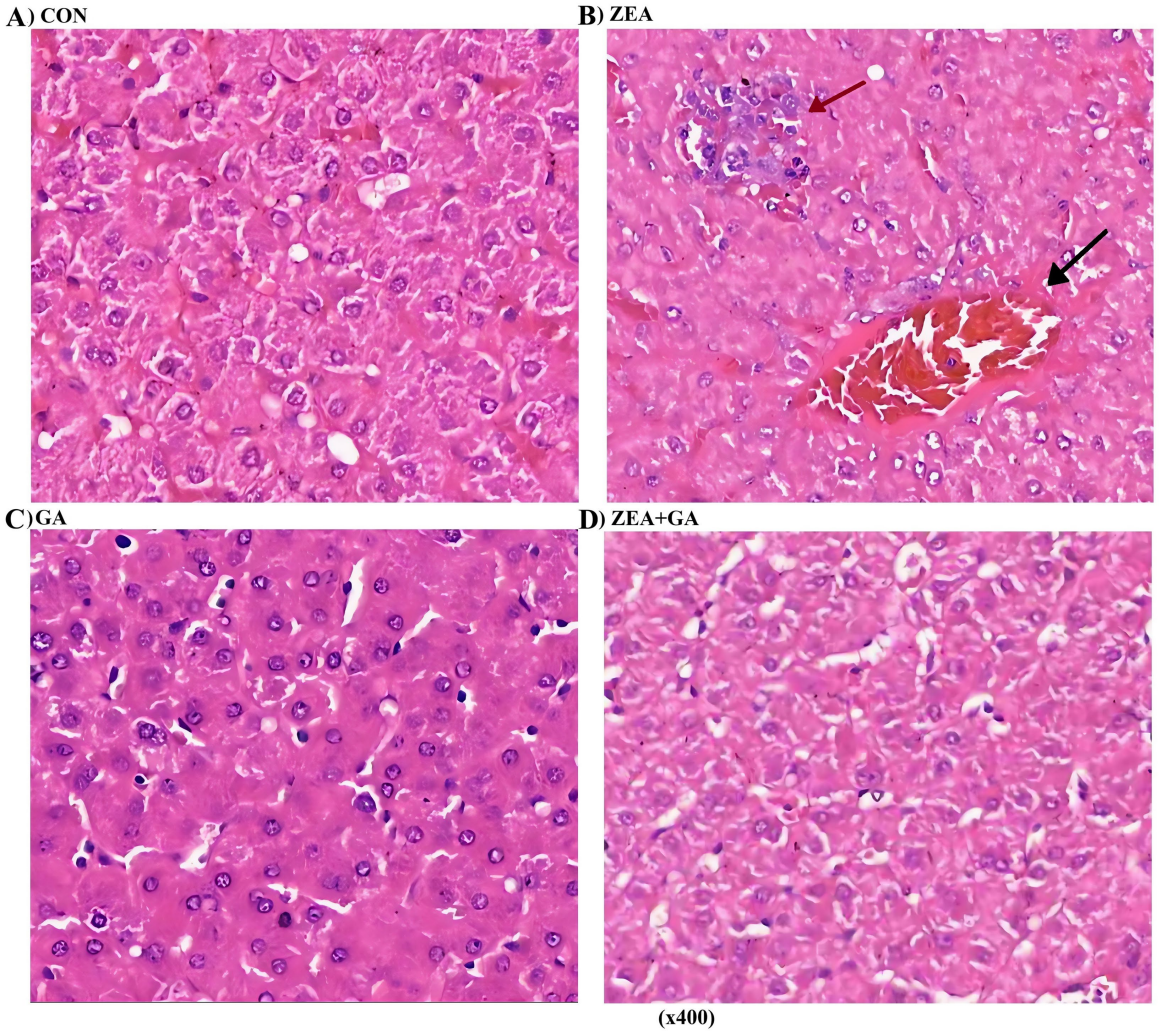
Effect of ZEN and GA on liver of replacement gilts: **(A,C)** No significant histological abnormalities were observed in the liver tissue; **(B)** Dilated sinusoids (↑) with congestion and focal hepatocyte necrosis (↑) surrounded by inflammatory infiltrates. **(D)** Cytoplasmic vacuolization was observed in hepatocytes.

As shown in Figure 3, the CON group exhibited intact hepatic capsules and normal hepatocyte morphology. In the ZEA group, the liver tissue maintained an intact capsule, but hepatocytes displayed focal necrosis accompanied by inflammatory cell infiltration (primarily lymphocytes). Additionally, hepatic sinusoids were slightly dilated with evident congestion. The GA group showed intact liver capsules and normal hepatocyte architecture, like the CON group. For the ZEA + GA co-treatment group, the hepatic capsule remained intact, though mild vacuolar degeneration was observed in hepatocytes. Overall, the liver cell morphology appeared normal.

### 3.7 The effect of ZEN and GA on HSD gene expression

**Figure 4:**
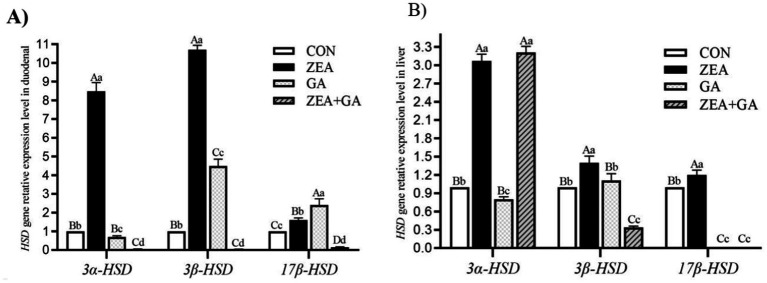
Effect of *ZEN* and GA on *HSD* gene relative expression level in the duodenum **(A)** and liver **(B)** of replacement gilts. In the same row, values with different small letter superscripts indicate a significant difference (*p* < 0.05), while those with different capital letter superscripts indicate a highly significant difference (*p* < 0.01).

The effect of ZEN and GA on HSD gene expression was presented in Figure 4 and Table 6. Analysis of the main effects revealed that dietary ZEA supplementation exerted highly significant effects (*p* < 0.01) on the relative mRNA expression levels of *3α-HSD*, *3β-HSD*, and *17β-HSD* genes in both the duodenum and liver of replacement gilts. Dietary GA supplementation significantly affected the relative mRNA expression of *3α-HSD* and *3β-HSD* genes in the duodenum, as well as *3β-HSD* and *17β-HSD* genes in the liver (*p* < 0.01). The interaction between ZEA and GA demonstrated highly significant effects (*p* < 0.01) on the relative mRNA expression of *3α-HSD*, *3β-HSD*, and *17β-HSD* genes in the duodenum, and *3β-HSD* and *17β-HSD* genes in the liver, while exerting a significant effect (*p* < 0.05) on *3α-HSD* expression in the liver.

**Table 6:** Effects of ZEN and GA *HSD* gene relative expression level in the duodenum and liver of replacement gilts (*n* = 5/group).

Parameter		*HSD* gene relative expression level in duodenum			*HSD* gene relative expression level in liver		
		*3α-HSD gene*	*3β-HSD gene*	*17β-HSD gene*	*3α-HSD gene*	*3β-HSD gene*	*17β-HSD gene*
ZEA Adding Levels, mg/kg	0	0.85 ± 0.16^Bb^	2.75 ± 1.86^Bb^	1.71 ± 0.78^Aa^	0.9 ± 0.11^Bb^	1.06 ± 0.09^Aa^	0.5 ± 0.53^Bb^
	1	4.26 ± 4.52^Aa^	5.37 ± 5.63^Aa^	0.88 ± 0.77^Bb^	3.14 ± 0.2^Aa^	0.87 ± 0.56^Bb^	0.6 ± 0.63^Aa^
GA, Adding Levels, mg/kg	0	4.74 ± 4.03^Aa^	5.85 ± 5.11^Aa^	1.3 ± 0.32	2.04 ± 1.1	1.2 ± 0.22^Aa^	1.1 ± 0.12^Aa^
	400	0.37 ± 0.35^Bb^	2.26 ± 2.36^Bb^	1.28 ± 1.21	2.01 ± 1.28	0.73 ± 0.41^Bb^	0 ± 0^Bb^
*p* value	ZEA	<0.001	<0.001	<0.001	<0.001	<0.001	<0.001
	GA	<0.001	<0.001	0.801	0.638	<0.001	<0.001
	ZEA + GA	<0.001	<0.001	<0.001	0.015	<0.001	<0.001

(1) Weight on the 19th day of the second estrus period. (2) In the same column, values with different small letter superscripts mean significant difference (*p* < 0.05), while values with different capital letter superscripts mean highly significant difference (*p* < 0.01). The same or no letter superscripts mean no significant difference (*p* > 0.05).

As shown in Figure 4A, compared with control, the relative expression level of *3α-HSD*, *3β-HSD,* and *17β-HSD* genes in the duodenum of the ZEA group was significantly increased (*p* < 0.01), and the *3α-HSD* gene was significantly decreased (*p* < 0.05) in the GA group. The relative expression level of the *3α-HSD* gene in the duodenum of the GA group was significantly reduced (*p* < 0.05). In contrast, the relative expression levels of the *3β-HSD* and *17β-HSD* genes were significantly increased (*p* < 0.01), but both were significantly lower than those in the ZEA group (*p* < 0.01). The *3α-HSD*, *3β-HSD*, and *17β-HSD* genes in the duodenum of the ZEA + GA group were significantly reduced (*p* < 0.01).

As shown in Figure 4B, compared with control, the relative expression level of *3α-HSD*, *3β-HSD* and *17β-HSD* genes in the liver of the ZEA group was significantly increased (*p* < 0.01). In contrast, the relative expression levels of *3α-HSD* genes in the GA group were significantly decreased (*p* < 0.05), and *17β-HSD* genes were significantly decreased (*p* < 0.01). The expression levels of *3α-HSD* genes in the liver of the ZEA + GA group were significantly increased (*p* < 0.01), while the relative expression levels of *3β-HSD* and *17β-HSD* genes were significantly decreased (*p* < 0.01).

## 4 Discussion

### 4.1 The impact of ZEN and GA on estrus onset in replacement gilts

This study provided more evidence that ZEN regulates estrus and puberty in gilts and that these effects are dose-dependent. Taking 1 mg/kg ZEN supplements accelerated the onset of puberty in terms of both weight and age, but it also caused irregular periods and a lengthening of the estrus interval. In line with previous research, this supports the idea that modest doses of ZEN can promote early estrus but frequently disturb the stability of the cycle, and that greater dosages delay or lengthen intervals even more (11, 18, 19). Contrarily, supplementing with GA at a dose of 400 mg/kg accelerated the start of estrus without changing the interval, indicating that GA enhances reproductive preparation without disrupting cyclicity. The fact that ZEN and GA, when given together, accelerated puberty to the same extent as ZEN alone while keeping cycle length normal, shows that GA protects against mycotoxin-induced reproductive disruption. These findings add to what is already known about GA as an 11-HSD inhibitor that can sustain estrus physiology when exposed to ZEN, therefore enhancing glucocorticoid activity and growth (20, 21).

### 4.2 Regulation of serum reproductive hormones

Hormonal findings provided mechanical explanations for these alterations in reproduction. Studies have shown that ZEN can inhibit gonadotropins and estradiol. This is supported by the fact that ZEN boosted IGF-1 and kisspeptin while decreasing LH and E2 (16, 22). Ovulation and follicular maturation are both thrown off by these alterations. The hormonal environment for ovarian development was improved by supplementing with GA, which increased IGF-1, Kp, LH, and E2. By restoring IGF-1 and LH to near-control levels and keeping E2 steady, a combination of ZEN and GA suggests that GA mitigates the inhibition of the HPGA axis by ZEN. Liang et al. (23) and Wang (24) found that GA can normalize hormone levels in pathological conditions, including hyperprolactinemia and gastric ulceration, which is consistent with our findings. It seems that GA promotes early but physiologically balanced puberty by acting at both upstream regulators (IGF-1, Kp) and downstream gonadotropins.

### 4.3 Effects on uterine development

Supplementation with ZEN dramatically changed the uterine morphology. Symptoms of inappropriate estrogenic stimulation included an enlarged uterus, shorter horns, a thicker cervix, and fewer loops in gilts that were given ZEN. Endometritis, hyperplasia, and connective tissue degradation were found in previous investigations that followed ZEN exposure (25, 26). These pathological effects are in line with our results. On the other hand, using GA supplements led to longer horns with more loops, which may have stimulated uterine growth. Partial morphological recovery was observed in gilts given ZEN + GA, as their horn length grew and their uterine weight dropped, as compared to gilts given ZEN alone. These results are consistent with those of soy isoflavones, which were found to decrease the toxicity of ZEN in gilts (16). Therefore, GA seems to mitigate uterine hypertrophy and promote normal development patterns, thus counteracting ZEN’s estrogenic overstimulation (9, 27).

### 4.4 Ovarian development and follicle quality

Ovarian morphology was a good indicator of how well a treatment was working. Follicle atrophy, discolouration, and decreased ovarian weight were observed in gilts that were fed ZEN. Follicle numbers for large, medium, and mature follicles were also lower. Similar results have been reported in the past by Chen et al. (28), Schoevers et al. (29), and Xiao (22), showing that ZEN hinders folliculogenesis, causes oocyte depletion, and induces cyst development. Ovarian weight and follicle counts were both raised by GA supplementation alone, which is in line with its known capacity to enhance oocyte quality and decrease ovarian tissue apoptosis (30–32). The ZEN + GA group demonstrated some improvement in follicle number and shape, which indicates that GA can mitigate the follicular toxicity caused by ZEN. There is some evidence that GA could be used as a reproductive enhancer in swine feed due to its protective effects against atresia and stimulatory effects for good folliculogenesis.

### 4.5 Effects on liver histology

Hepatocyte necrosis, sinusoidal stasis, and leukocyte infiltration were all symptoms of liver damage caused by ZEN supplementation, as one would predict. Long et al. (33) and Wang (16) have provided ample evidence of these hepatotoxic effects in rats and pigs. With GA supplementation, liver histology was conserved, and lesions were significantly decreased in the ZEN + GA group. It is consistent with other research that has shown that GA can protect the liver by acting on three different pathways: antioxidant, anti-inflammatory, and anti-fibrotic (34, 35). Livestock production, where animals often come into contact with dietary mycotoxins, is very pertinent to the protection afforded by GA, as the liver plays a crucial role in xenobiotic metabolism.

### 4.6 Modulation of HSD gene expression and overall implications

Based on molecular investigation, ZEN enhanced conversion to more estrogenic metabolites by dramatically upregulating 3α-HSD, 3β-HSD, and 17β-HSD in both the gut and the liver (4, 7, 8). The observed impairment of endocrine functions and tissue damage could be explained by this metabolic change. These enzymes were affected by GA supplementation, which decreased duodenal 3α-HSD and hepatic 17β-HSD. In the ZEN + GA group, 3ο- and 17Ų-HSD were dramatically downregulated, whereas hepatic 3α-HSD was modestly increased. These changes indicate that GA inhibits important enzymes involved in the production of estrogenic metabolites, which in turn interferes with the bioactivation of ZEN (10). Similar interactions have been shown in pigs when soy isoflavones are used to reduce the increase in HSD caused by ZEN (16, 36).

Taken together, our results show that replacement gilts exposed to low dietary amounts of ZEN nonetheless experience liver impairment, altered endocrine signaling, damaged uterine and ovarian development, and disrupted estrus cycles. Consistent with previous toxicological investigations showing long-term effects on reproduction following ZEN exposure, these results support the findings of Gajecka et al. (37), Soffa et al. (38), and Liu et al. (39). The negative effects of these outcomes were reduced by taking GA supplements in a multi-systemic way. First, it stabilized the reproductive cycles and the start of puberty. Second, it restored hormonal balance in the HPGA axis. Third, it reduced structural abnormalities in the uterus and the ovaries. Fourth, it protected the liver. Finally, it modulated *HSD* gene expression to decrease ZEN bioactivation. To protect gilts’ reproductive performance when exposed to mycotoxins, GA has two purposes, which make it an attractive dietary intervention.

Additional research is needed to determine the relationship between dose and response, measure the tissue concentrations of ZEN metabolites, and explore the mechanisms at the receptor and pathway levels; this study only employed single doses of ZEN and GA. The scope of GA’s preventive potential can be better understood, and its practical uses in pig production can be better informed by such investigations. Although mycotoxin contamination is inevitable in livestock management, the current results show that GA supplementation is a viable technique to reduce ZEN toxicity in replacement gilts. This strategy may find broader use in other contexts as well.

## 5 Conclusion

Supplementation with GA in the diet of replacement gilts can regulate their reproductive hormone levels, promote the development of their uterus and ovaries, mitigate ZEA’s hepatotoxic effects, and regulate the expression levels of *HSD* genes in the intestine and liver. GA can significantly reduce the adverse effects of ZEN on the reproductive physiology of replacement gilts, thereby alleviating its reproductive harm. However, reproductive function is regulated by complex endocrine systems and elements of nutritional metabolism. Due to the limited understanding of the metabolic process of ZEN and the mechanism of action of GA in humans, further study is needed to elucidate the mechanism of GA and its regulatory effects on ZEN.

## Data Availability

The original contributions presented in the study are included in the article/supplementary material, further inquiries can be directed to the corresponding author.
